# Experimental detection of the diamino-pentazolium cation and theoretical exploration of derived high energy materials

**DOI:** 10.1038/s41598-024-60741-z

**Published:** 2024-05-02

**Authors:** Tianyang Hou, Xiaofeng Yuan, Shuaijie Jiang, Ze Xu, Xiaopeng Zhang, Ming Lu, Yuangang Xu

**Affiliations:** School of Chemistry and Chemical Engineering, Nanjing University of Science and Technology, Nanjing, 210094 China

**Keywords:** Inorganic chemistry, Physical chemistry, Chemical synthesis, Chemistry, Materials science

## Abstract

In this work, we realized the detection of diamino-pentazolium cation (DAPZ^+^) in the reaction solution experimentally and proved it to be meta-diamino-pentazole based on the transition state theory. Quantum chemical methods were used to predict its spectral properties, charge distribution, stability and aromaticity. Considering that DAPZ^+^ has excellent detonation properties, it was further explored by assembling it with N_5_^−^, N_3_^−^ and C(NO_2_)_3_^−^ anions, respectively. The results show a strong interaction between DAPZ^+^ and the three anions, which will have a positive effect on its stability. Thanks to the high enthalpy of formation and density, the calculated detonation properties of the three systems are exciting, especially [DAPZ^+^][N_5_^−^] (*D*: 10,016 m·s^−1^; *P*: 37.94 GPa), whose actual detonation velocity may very likely exceed CL-20 (*D*: 9773 m·s^−1^). There is no doubt that this work will become the precursor for the theoretical exploration of new polynitrogen ionic compounds.

## Introduction

Polynitrogen compounds (containing only nitrogen atoms), also known as nitrogen allotropes, are promising candidates as high-energy–density materials (HEDMs)^1–3^. The high energy content of polynitrogen compounds is due to the significant difference in bond energy between nitrogen atoms^4^. Especially nitrogen atoms in polynitrogen compounds are connected through single and double bonds (N=N double bond energy is 418.7 kJ·mol^−1^, N–N single bond energy is only 160.8 kJ·mol^−1^), while the N≡N triple bond energy in nitrogen gas (N_2_) as the decomposition/detonation product is characterized as a record-breaking value of 958.8 kJ·mol^−1^ ^5^. Compared with traditional energetic materials, polynitrogen compounds have theoretical advantages such as sufficiently high densities, uniquely high heats of formation, ultra-high energy levels, and environmentally friendly decomposition products. Therefore, polynitrogen compounds are expected to have broad application prospects in fields such as aerospace, weapons and equipment.

**Figure 1:**
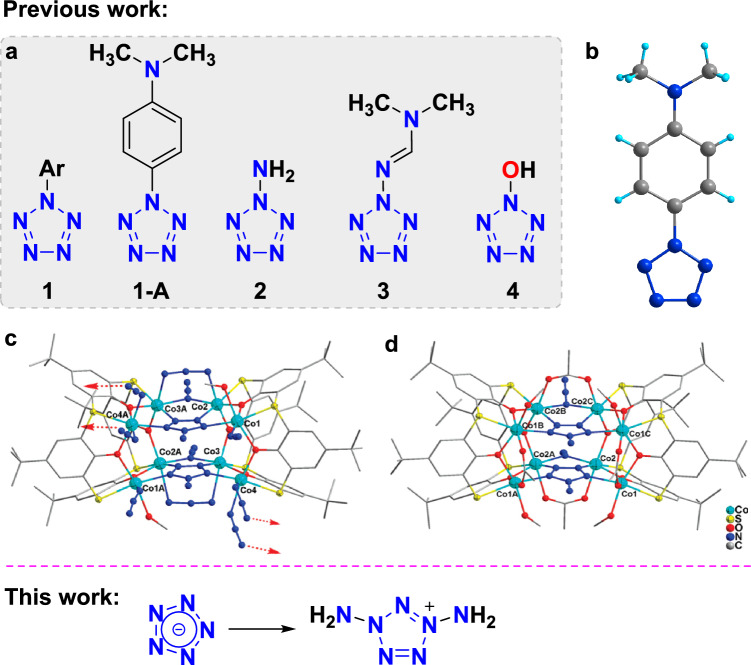
Covalent derivatives of pentazole studied in experiments. **(a)** The structural formula of the covalent derivatives of pentazole. (**b**) Single-crystal structure of 4-dimethyl-aminophenylpentazole. (**c**) Single-crystal structure of [Co_4_(TC4A)(N_3_)_4_(N_6_H_2_)(CH_3_OH)](CH_3_OH)_2_. (**d**) Single-crystal structure of [Co_8_(TC4A)_2_(N_3_)_2_(N_6_H_2_)_2_(CH_3_COO)_4_(CH_3_OH)_4_](OH)_2_(CH_3_OH)_4_.

Pentazolate anion (*cyclo*-N_5_^−^), a cyclic polynitrogen species composed of five nitrogen atoms, has received increased attention since its bulk synthesis in 2017^6,7^. Great progress has been made in the creation of its metallic salts, coordination compounds, organic salts, and cocrystals^8–12^. However, these compounds are all ionic derivatives of pentazole. There is very little experimental exploration of its covalent derivatives. In fact, the first report with evidence of the covalent derivatives of pentazole was the arylpentazole (**1**, Fig. 1a) which can be date back to 1956^13–15^. Later, isolation and even single-crystal X-ray diffraction analysis of the arylpentazole (4-dimethyl-aminophenylpentazole, **1-A,** Fig. 1b) were successful^16–18^. However, attempted modifications of the aryl group have usually resulted in destruction of the pentazole ring, which degraded rapidly at ambient temperature with evolution of N_2_^19,20^. Those relatively stable arylpentazoles are commonly used as precursors for cleaving C–N bonds to obtain *cyclo*-N_5_^−^^21,22^.

In 2010, aminopentazole (N_6_H_2_, **2**), as a single-ring pentazole derivative based on covalent bonding was identified by Liao and Zhang et al*.*^23^. However, the N_6_H_2_ molecules, which serves as the ligands formed in situ of cobalt clusters [Co_4_(TC4A)(N_3_)_4_(N_6_H_2_)(CH_3_OH)](CH_3_OH)_2_ (Fig. 1c) and [Co_8_(TC4A)_2_(N_3_)_2_(N_6_H_2_)_2_(CH_3_COO)_4_(CH_3_OH)_4_](OH)_2_(CH_3_OH)_4_ (Fig. 1d), had not been isolated for further research. Another covalent derivative of pentazole is N_5_-N=CH–N(CH_3_)_2_ (**3**), which was synthesized by Banert et al. in 2020^24^. This aminopentazole derivative was generated in a mixture with N_3_–N = CH–N(CH_3_)_2_ in a molar ratio of approximately 2:1, and their identification was mainly based on NMR spectroscopy, especially ^15^N NMR data. Unfortunately, the half-life of the aminopentazole derivative in *d*_7_-DMF solution was estimated to be around only 11 min at 21 °C. Very recently, Lu and Xu et al*.* attempted to use Oxone to oxidize *cyclo*-N_5_^−^ to obtain covalent derivatives N_5_OH (**4**) or N_5_O^−^^25^. However, due to a very high activation energy barrier involved in the reaction, they were unable to obtain the expected pentazolate *N*-oxide. Overall, despite the tremendous efforts of researchers from around the world, the syntheses of covalent pentazole derivatives remain a significant challenge due to their poor stabilities.

In our continued efforts to explore pentazole derivatives, we tend to use *cyclo*-N_5_^-^ ionic derivatives as precursors for the construction of covalent bonds on the pentagonal ring. Fortunately, it is proud that in this work, based on our previous research, the diamino-pentazolium cation (DAPZ^+^) was successfully prepared and characterized, and the related contents were supplemented by the theoretical calculation method of quantum chemistry. Considering its high energy, the assembled ionic compound system was analyzed in detail by Atoms in Molecules (AIM), Independent Gradient Model based on Hirshfeld Partition (IGMH), Electrostatic Potential (ESP) and Symmetry-Adapted Perturbation Theory (SAPT), and the detonation performance was also taken into account.

## Results

### Synthesis and reaction mechanism of ***m***-DAPZ^+^

With [Na(H_2_O)(N_5_)]·2H_2_O^6^ in hand, we focused on the corresponding *N*-amination reaction using *O*-*p*-toluenesulfonylhydroxylamine (THA) and HOSA. However, the *N*-amino product could be prepared only by HOSA instead of THA.

The amination of *cyclo*-N_5_^−^ was carried out at 30, 40, and 50 °C, respectively, and an interesting phenomenon was found. The amination of *cyclo*-N_5_^−^ was not successful at 30 °C, and the mass spectra showed that the amination reagent and the *cyclo*-N_5_^−^ in the reaction mixture. At 40 °C, obvious peaks of a diamino-substituted pentazole species were observed at *m/z* = 100.945/102.128 and mass-selected for subsequent MS/MS studies. No amination product was generated in the reaction system at 50 °C, and only the signal peaks of the amination reagents were present in the mass spectra, which may be related to the decomposition of *cyclo*-N_5_^-^ precedes the reaction. Except for the signal of 100.945, HSO_4_^−^, and SO_3_^2−^ were confirmed by secondary mass spectrometry to be by-products of amination reagents HOSA (Fig. S1).

**Figure 2:**
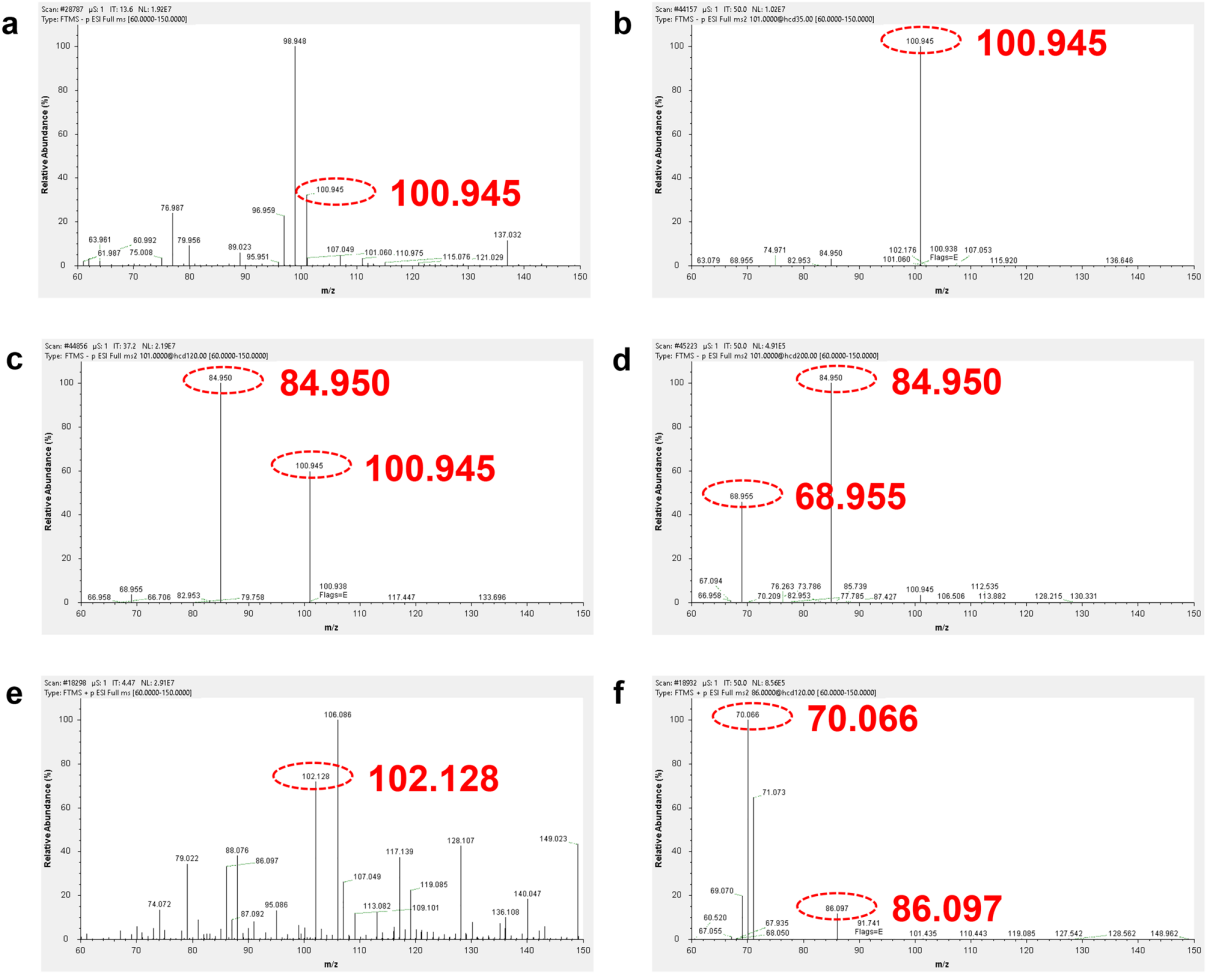
High-resolution mass spectra of the reaction solution. **(a)** Negative ion, mass spectrum of the reaction solution at 40 °C for 8 h. (**b–d**) Negative ion, full-range CID mass spectra of the mass selected, (*m/z* 100.945) peak recorded at collision voltages of −30, −120, and −200 V. (**e**) Positive ion, mass spectra of the reaction solution at 40 °C for 8 h. **f** positive ion, full-range CID mass spectrum of the mass selected, (*m/z* 102.128) peak recorded at collision voltages of 30 V.

Further confirmation was obtained by MS/MS analysis of the *m/z* = 100.945 peak in negative ion mode (Fig. 2a). Using a low collision voltage of 30 V, the *m/z* 100.945 ion was not shattered (Fig. 2b). As the collision voltage increased to 120 V, the *m/z* 100.945 ion underwent stepwise NH_2_ loss, giving rise to an intense peak with *m/z* value of 84.950 (Fig. 2c). Then the voltage continued to increase to 200 V, the peak of *m/z* = 100.945 almost disappeared and underwent stepwise second NH_2_ loss rise to an intense peak with *m/z* value of 68.955 (Fig. 2d). According to the mass spectrum in positive ion mode, the peak of *m/z* = 102.128 was detected, the *m/z* = 86.097 and 70.066 were obtained by MS/MS analysis using a low collision voltage of 30 V which underwent twice NH_2_ (Fig. 2e, f).

The pentazolate species replaced by two amino groups (DAPZ) cannot be identified from the mass spectrum as positive ion, negative ion or neutral molecule (Fig. S2). Therefore, different structures of DAPZ, DAPZ^-^, and DAPZ^+^ were optimized by Gaussian09 at the M06-2X/6-31+G(d,p) level. However, it was impossible to calculate neutral DAPZ molecules (53 electrons) with multiplicity 1. For the negative ion state, the optimized configurations of the five DAPZ^-^ ions obtained are not reasonable (Fig. S3). Finally, only the positive ion state is suitable. The five optimized DAPZ^+^ structures having no imaginary frequencies and their total energies, enthalpies, free energies are shown in Fig. S4. Two of them are *o*-DAPZ^+^ (**o-1**, **o-2**), and the other three are *m*-DAPZ^+^ (***m-1–*** ***m-3***). We can find two pairs of chiral isomers with equal energies. According to the principle of minimum energy, the possible structure should be the bottom two *m*-DAPZ^+^ (chiral isomers) in Fig. S4.

**Figure 3:**
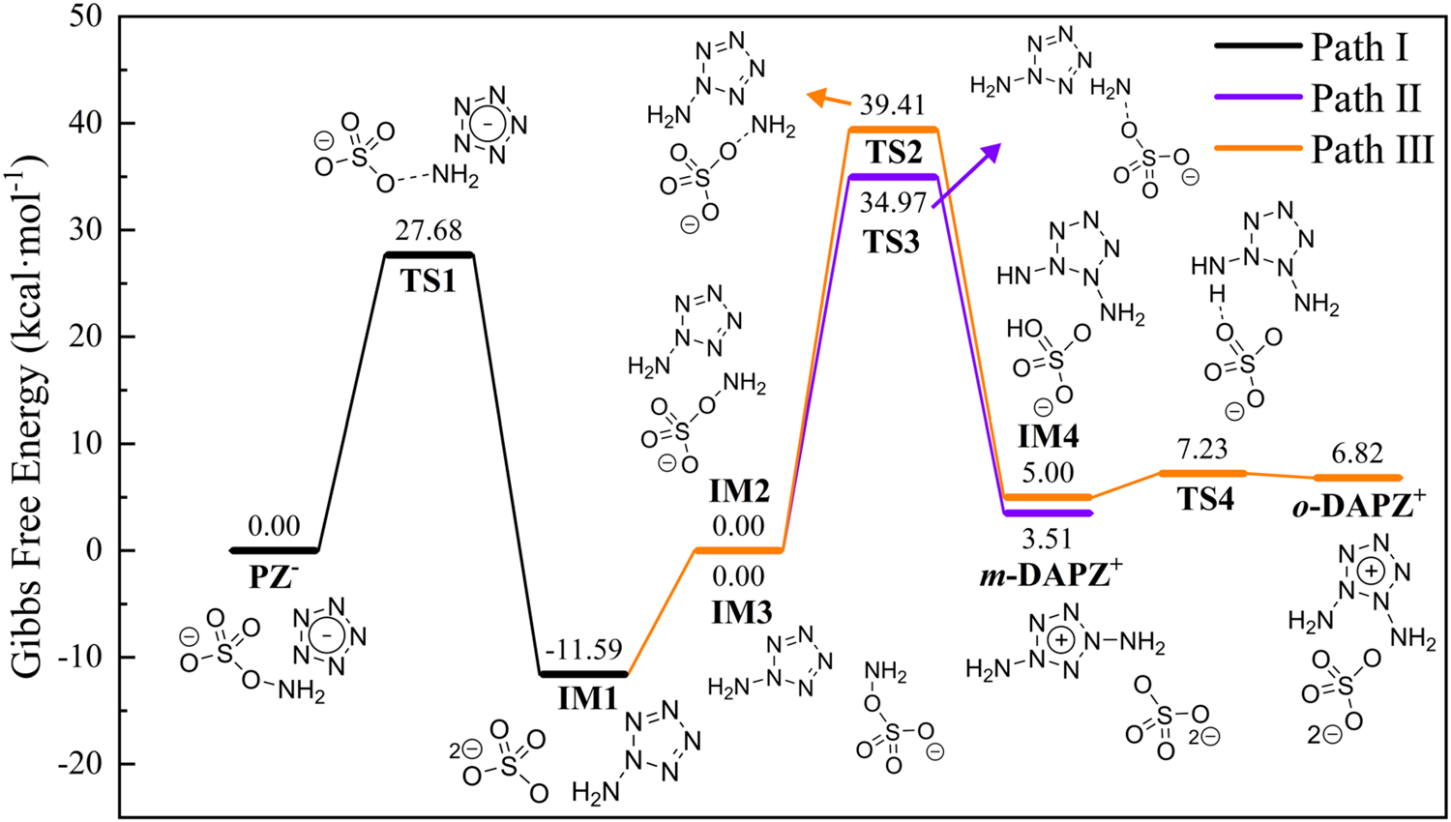
Amination mechanism and energy barrier of DAPZ^+^.

In order to further investigate the amination process and product structure in detail, the transition state of the whole process is calculated, as shown in Fig. 3, where IM refers to the intermediate substance and TS refers to the transition state found by different paths. Path I–III refers to pentazole amination, amino pentazole meta-amination and ortho-amination respectively, and the whole reaction path uses relative Gibbs free energy. It should be noted that the relative energies of the initial reactants of these three reaction paths are all set to 0, but their absolute Gibbs free energies are different, so as to compare the reaction barriers of different paths. The intrinsic reaction coordinate (IRC) calculations were performed on the details of the amination process, re-disassembled and integrated the process in which the transition state reaches the reactant and the product from two directions, obtaining a complete reaction process from reactants to transition state and then to products. Please refer to the MP4 file in the supporting information. There are four videos provided, which correspond to the IRC processes of four reaction transition states, and more calculation details are in the following sections.

In the process of amination of *cyclo*-N_5_^−^, the potential barrier of TS1 is 27.68 kcal·mol^−1^, and the Gibbs free energy of the intermediate product IM1, amino-pentazole, is lower than that of *cyclo*-N_5_^−^, which means that the amino-pentazole system is relatively more stable than that of *cyclo*-N_5_^−^. IM1, was further aminated, and the initial states of ortho and meta-amination were IM2 and IM3 respectively. Two different transition state paths show completely different information. From the distribution of transition states, the transition state of ortho-amination TS2 is above the transition state of meta-amination TS3. It means that the barrier of ortho-amination will be higher and the reaction will be more difficult, which is confirmed by the comparison of Gibbs free energy. In addition, it is worth noting that in the process of ortho-amination, it can be seen that amino-pentazole cannot form *m*-DAPZ^+^ in one step, but rather undergoes a hydrogen transfer process. However, the energy required for hydrogen transfer from the intermediate IM4 to *o*-DAPZ^+^ is only 2.23 kcal·mol^−1^, and the energy of the transition state of the reverse reaction is even less than 0.41 kcal·mol^−1^, so *o*-DAPZ^+^ will be easily converted into IM4. In other words, *o*-DAPZ^+^ will be difficult to exist stably in the system. Another important phenomenon is that the final energy of *m*-DAPZ^+^ will be lower than that of *o*-DAPZ^+^, which indicates that *m*-DAPZ^+^ will be more stable in the system. After the above analysis, there is no doubt that most of the synthesized product is *m*-DAPZ^+^, but due to the technical limitations and the difficulty of synthesis, a very small amount of *o*-DAPZ^+^ will probably exist in the whole system in the form of "impurities".

Positively charged ***m-2*** (or ***m-3***) is considered the most likely amination product, which has C_2_ symmetry (Fig. S5). The bond lengths, bond angles, and torsion angles of ***m-2*** are presented in Tables S1–S3. It consists of a five membered ring with a nearly planar structure and two meta-amino groups. The two amino groups are slightly deviated from the ring based on the torsion angles of 173.66° (N4–N3–N7–N11 and N8–N11–N7–N3). In the five-membered ring, the bond lengths of N1–N2, N1–N3 (or N2–N11) and N3–N7 (or N11–N7) are 1.305, 1.322 and 1.314 Å, respectively, which are shorter than that of the N–N (1.450 Å), implying they have properties of partial double bonds. The N-NH_2_ bond (1.359 Å) is the longest N–N bond in the molecule, but it is also shorter than the conventional N–N bond. For the bond angles, ***m-2*** is not regular polygon structures, and the bond angles are in the range of 100.33°–114.17°.

**Figure 4:**
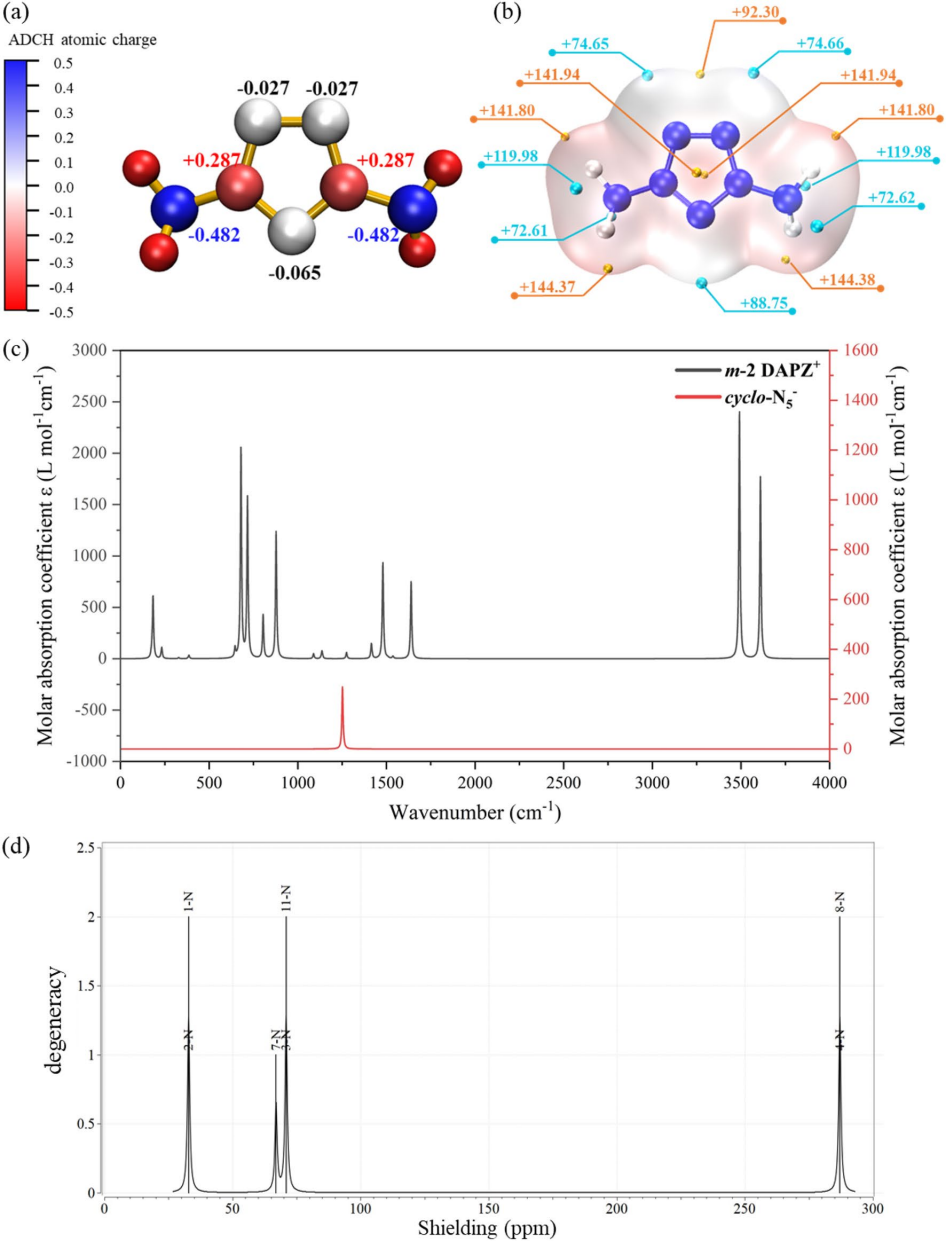
Theoretical structural characterization of ***m-2***. **(a)** The ADCH charges of ***m-2***. (**b**) Molecular ESP of ***m-2***. (**c**) The calculated IR spectra of ***m-2*** and *cyclo*-N_5_^−^. (**d**) The calculated ^15^N NMR spectrum of ***m-2***.

Atomic charge is one of the simplest and most intuitive forms of description of charge distribution in chemical systems and is important for the analysis of both chemical reactions and adsorption principles. Hirshfeld charge can be rectified using atomic dipole moment corrected Hirshfeld (ADCH charge), as the Hirshfeld charge levels are typically tiny and the dipole moment and electrostatic potential repeatability are quite low. In calculating the ADCH charge, the Hirshfeld charge and atomic dipole moment of each atom are first calculated, and then each atomic dipole moment is expanded into the corrected charge of the surrounding atoms, and sum them to obtain the ADCH charge. The charges of each atom in ***m-2*** under the solvent model are shown in Fig. 4a. The charges on N3 and N11 of ***m-2*** are more than those in N1, N2 and N7 because both N3 and N11 are connected to the electron-donating amino groups, which makes the charges on the amino group transfer to the adjacent nitrogen atoms on the ring. In addition, the nitrogen atom on the amino group transfers some of its charge to the hydrogen atoms, resulting in a reduction in its own charge, making the entire molecule more evenly charged and stable. Molecular electrostatic potential (ESP) of ***m-2*** is analyzed by the Multiwfn program and shown in Fig. 4b. The maximum and minimum ESP values of ***m-2*** are 144.38 kcal·mol^−1^ and 72.61 kcal·mol^−1^, respectively. Moreover, the electropositive occupied the whole molecule. There is a positive electrode value point on both sides of the molecular center, with a value of 141.94 kcal·mol^−1^.

In order to provide experimentalists with spectral data for further instrumental identification of DAPZ^+^ cation, we had calculated IR (Fig. 4c) and NMR (Fig. 4d) spectra of ***m-2***. Obviously, the absorptions of ***m-2*** are more complex than the *cyclo*-N_5_^−^, the new absorptions at 180, 706, 841, and 1654 cm^−1^ are attributed to the torsional vibrations of the N–H bonds. The strongest absorption at 3507 and 3628 cm^−1^ are attributed to the N–H stretching vibration of the amino groups. The N–N stretching vibration of the N-amino groups (N3–N4 and N8–N11) and the ring (N1–N3 and N2–N11) are located at 765 and 1540 cm^−1^, respectively. The calculated ^15^N NMR spectrum using nitromethane (Fig. S6) as TMS shows signals for the five-membered ring are at 32.008 (N1 and N2), 66.153 (N7), and 70.088 ppm (N11 and N3). In addition, the N4 and N8 of amino groups are observed at 286.233 ppm (Fig. 4d).

The HOMO–LUMO orbitals of ***m-2*** and *cyclo*-N_5_^−^ were also studied. The energy levels and the energy gaps are given in Fig. S7. The red phase means positive, while the blue is negative. The energy gap is an important value that can evaluate whether the electrons can be excited easily or not. In some ways, larger energy gaps mean higher chemical stability. As presented in Fig. S7, ***m-2*** has much higher energy gap (12.85 eV) than the *cyclo*-N_5_^−^ (11.82 eV), which indirectly indicates that ***m-2*** is more chemical stable than *cyclo*-N_5_^−^.

**Figure 5:**
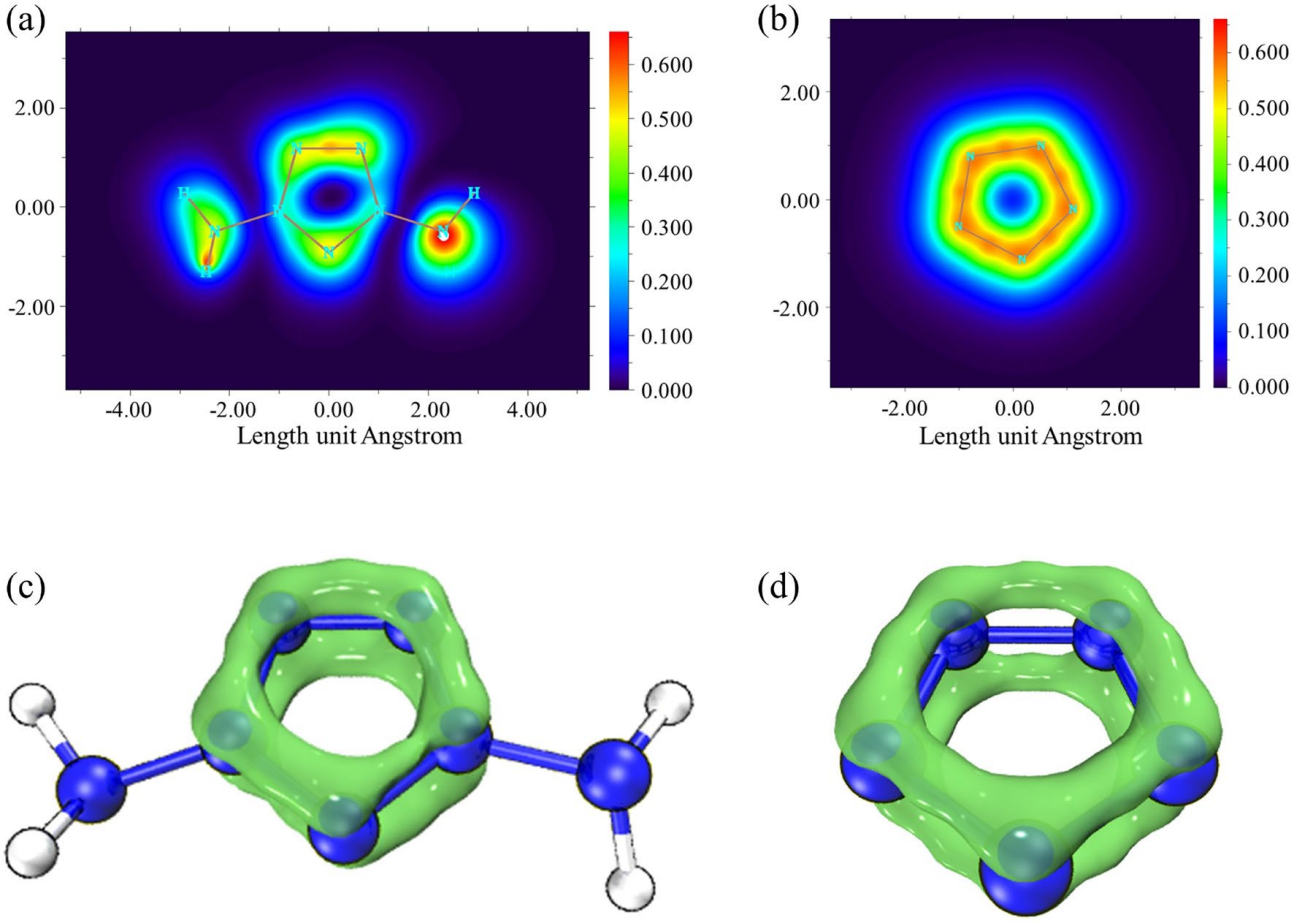
Pathway of *π* electron delocalization of ***m-2*. (a)** and *cyclo*-N_5_^−^, (**b**) (1.2 Bohr above the XY plane). The *π* electron density distribution diagram of ***m-2*** **(c)** and *cyclo*-N_5_^−^ (**d**) at the isosurface of 0.4.

In addition, the delocalized electron clouds of ***m-2*** and *cyclo*-N_5_^−^ were visualized by local orbital localizer (LOL) analysis (Fig. 5a, b). In the π-electron density plane, the colors from dark blue to red show the low–high trend of π-electron density. Due to the influence of two amino groups, the aromaticity of ***m-2*** is less than that of *cyclo*-N_5_^−^. Similar to *cyclo*-N_5_^−^, but smaller number of electrons gather on the five-membered ring, forming an almost closed electron ring in ***m-2*** (Fig. 5c, d). Table S4 shows that the calculated bond dissociation energy (BDE) of ***m-2*** is 342.82 kJ·mol^−1^ at 298.15 K. The large BDE indicates that once ***m-2*** is synthesized, it will have a good stability, either spectroscopically or even in the condensed phase.

Finally, the thermal stability of DAPZ^ +^ must be mentioned. In fact, this is a common problem of all covalent pentazole derivatives, mainly because various types of chemical bonds formed between N atoms are easy to break and decompose under external stimulation, despite their high bond energy. Recently, a good approach was developed to estimate thermal stabilities of covalent and ionic pentazole derivatives using the longest N–N bond length in the ring^26^. The general conclusion is that the thermal decomposition temperature decreases with the increase of the longest N–N bond length. At the level of M06-2X/6-31+G(d,p), the bond length of the longest N–N in *cyclo*-N_5_^−^ after optimization is 1.326 Å, while that in DAPZ^ + ^ is 1.359 Å. It can be inferred that the thermal decomposition temperature of DAPZ^ + ^ will be lower than that of *cyclo*-N_5_^−^. Further, it is not difficult to imagine that the synthesis and isolation of DAPZ^ + ^ is very difficult. Fortunately, this instability can be alleviated by effectively enhancing the interaction between ions through hydrogen bonds, *π* bonds, and conjugated structures, thereby improving the stability of the whole system. Therefore, selecting appropriate anions is another part of this work.

### Interaction analysis of assembled ion system

#### AIM analysis

**Figure 6:**
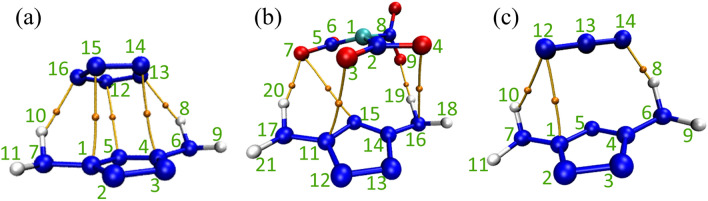
Electron density topological diagrams of three ion systems (orange spheres represented bond critical points (BCPs). Bond paths were drawn as orange paths).

The theory of atoms in molecules (AIM) plays an important role in studying the interaction between atoms^27^. Topological analysis of electron density is a key part of AIM framework. The point in space with the first derivative of the vanishing electron density is called the critical point (CP). According to the sign of eigenvalues of electron density Hessian matrix, it can be divided into four categories, namely nuclear critical point (NCP), bond critical point (BCP), ring critical point (RCP) and cage critical point (CCP)^28,29^. Among them, BCP has attracted extensive attention because it can reflect the interaction between atoms. Figure 6 shows the electron density topology of three ionic compound systems, and the external diameters are also given^30^. Some key topological properties of these BCPs are listed in Table 1.

**Table 1:** Electron density topological parameters at BCPs.

Composition	Interaction	*ρ*(*r*)	∇^2^*ρ*(*r*)	*H*(*r*)	*G*(*r*)	*V*(*r*)
[*m-*DAPZ^+^][N_5_^−^]	H10…N16	0.021612	0.066946	0.000693	0.016043	−0.01535
	N1…N15	0.014482	0.057853	0.001619	0.012845	−0.01123
	N5…N12	0.011773	0.051187	0.002016	0.010781	−0.00876
	N4…N14	0.01448	0.057844	0.001619	0.012842	−0.01122
	H8…N13	0.021615	0.066953	0.000693	0.016045	−0.01535
[*m-*DAPZ^+^][C(NO_2_)_3_^−^]	O4…N16	0.010523	0.045432	0.001944	0.009414	−0.00747
	H19…O9	0.028512	0.093895	−0.001129	0.024603	−0.02573
	O3…N11	0.014581	0.065069	0.002176	0.014091	−0.01192
	O7…N15	0.007506	0.032746	0.001727	0.006459	−0.00473
	H20…O7	0.030077	0.104244	−0.000936	0.026997	−0.02793
[*m-*DAPZ^+^][N_3_^−^]	H8…N14	0.06311	0.059106	−0.021872	0.036648	−0.05852
	N1…N12	0.016838	0.063661	0.001263	0.014653	−0.01339
	H10…N12	0.020107	0.068567	0.001421	0.015721	−0.01430

Generally speaking, the Laplace operator of electron density (∇^2^*ρ*(*r*)) can reflect the type of interaction. When ∇^2^*ρ* > 0, it indicates the existence of closed shell interaction (i.e. hydrogen bond and van der Waals interaction)^31^. Therefore, it can be clearly seen from the figure and table that there is interaction between some N and O atoms in the three systems. On the basis of Popelie's criterion for judging the existence of hydrogen bonds, Rozas further constructed a criterion for judging the strength of hydrogen bonds, that is, when ∇^2^*ρ* > 0 and *H*(*r*) > 0, hydrogen bonds are weak; When ∇^2^*ρ* > 0 and *H*(*r*) < 0, there is a moderate strength hydrogen bond. When ∇^2^*ρ* < 0 and *H*(*r*) < 0, the hydrogen bond strength is high^32,33^. From the data in Table 1, it can be seen that H atoms on *m-*DAPZ^+^ will form hydrogen bonds with anions in all three systems. The difference is that in the [*m-*DAPZ^+^][N_5_^−^] system, the strength of hydrogen bonds formed is weak, for example, the electron density between H10…N16 is only 0.021612 a.u., its ∇^2^*ρ*(*r*) is 0.066946, and *H*(*r*) is 0.000693, all of which are positive numbers. In the [*m-*DAPZ^+^][C(NO_2_)_3_^−^] system, all hydrogen bonds with moderate strength are formed. These hydrogen bonds will play a very important role in stabilizing the system and enhancing the mutual attraction between anion and cation. But It must be admitted that the attractive interaction between *m-*DAPZ^+^ and C(NO_2_)_3_^−^ will be stronger than that of N_5_^−^, and the system will be more stable. In addition, for the [*m-*DAPZ^+^][N_3_^−^] system, the first thing that can be determined is that weak hydrogen bonds must be formed between H10…N12. As for the interaction between H8…N14 atoms, although its atomic species conforms to the basic type of hydrogen bonding, its electronic density is 0.06311 a.u., which will not be within the scope of the existence standard of hydrogen bonding. It should be explained that the electron density between these two atoms is significantly higher than that between the atoms forming hydrogen bonds in the system, and there is no covalent bond between them, which means that not only hydrogen bonds may be formed between H8…N14, but also the strength of hydrogen bonds will be higher. More importantly, although the electron density at the critical point of hydrogen bonding in the three systems is higher, and the interaction intensity of hydrogen bonding is stronger than that between other atoms (probably stronger van der Waals interaction), the interaction between these atoms also plays an important role in the stability of the complex.

### IGMH and interaction energy analysis

**Figure 7:**
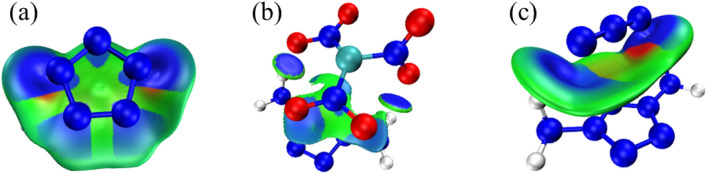
IGMH analysis of *m-*DAPZ^+^ (the sign(*λ*_2_ )*ρ* colored iso-surfaces of δg^inter^ = 0.003 a.u.).

Noncovalent interactions (NCI) analysis is a graphical method to study weak interaction, which can not only show the location of interaction directly, but also show the type and intensity of interaction at the same time^34,35^. Among them, by mapping the sign(*λ*_2_)*ρ* function with different colors, IGMH can not only clearly describe the types and properties of the interaction, but also flexibly customize the segments, and can only display the weak interaction between segments without interference from within the segments^36^. Therefore, in order to further explore the interaction between *m-*DAPZ^+^ and other anions, the above three systems were analyzed by IGMH, as shown in Fig. 7. Different colors represent different types of interactions. Blue represents weak attractive interactions, such as hydrogen bonds and halogen bonds; Green represents van der Waals interaction; Red represents repulsive interactions, such as steric effects in cages and rings.

First of all, in the system of [*m-*DAPZ^+^][N_5_^−^], it can be clearly seen that some N atoms on the N_5_^-^ anion will form hydrogen bonds with H atoms on *m-*DAPZ^+^, which is consistent with the analysis results of AIM. In addition, in AIM analysis, there will be strong interaction between some N atoms on anion and cation, which can be fully explained in Fig. 7a, N_5_^−^ and *m-*DAPZ^+^ contain typical conjugated *π* bonds. When they form a composite system, they will inevitably show the form of conjugated *π–π* stacking, which explains that blue appears around every N atom in N_5_^−^ in Fig. 7b. Secondly, other regions are mainly green, which means that the electron density is low, and there are extensive van der Waals interactions in this position. Among the three systems, the overall green region area of [*m-*DAPZ^+^][N_5_^−^] system is the largest. The difference is that in the [*m-*DAPZ^+^][N_3_^−^] ion system, in addition to the common hydrogen bonds, the N atom on N_3_^−^ will still form visible interaction with the N atom on *m-*DAPZ^+^. Thanks to the special electronic properties of N_3_^−^, in Fig. 7c, the N atoms in the anion and cation will form LP (lone pair)–*π* interaction^37^, which is very common in the analysis of weak interaction of energetic materials. It is worth noting that these interactions play a key role in the mutual attraction of composite systems. An interesting phenomenon is that there are some red areas in both systems, which is obviously normal, because even in the cationic system, some atoms will be negatively charged and show polarity. Therefore, it is not difficult to draw a conclusion that anions will repel the more polar atoms on *m-*DAPZ^+^. In fact, the negatively charged N atom on the amino group in *m-*DAPZ^+^ conforms to the above analysis and the position of the red area in Fig. 7 proves that this is the case. The IGMH of [*m-*DAPZ^+^][C(NO_2_)_3_^−^] system is similar to that of the other two systems. *m-*DAPZ^+^ will form strong hydrogen bonds with the active O atoms on it, and the van der Waals interaction between them will also stabilize the overall structure of [*m-*DAPZ^+^][C(NO_2_)_3_^−^].

**Figure 8:**
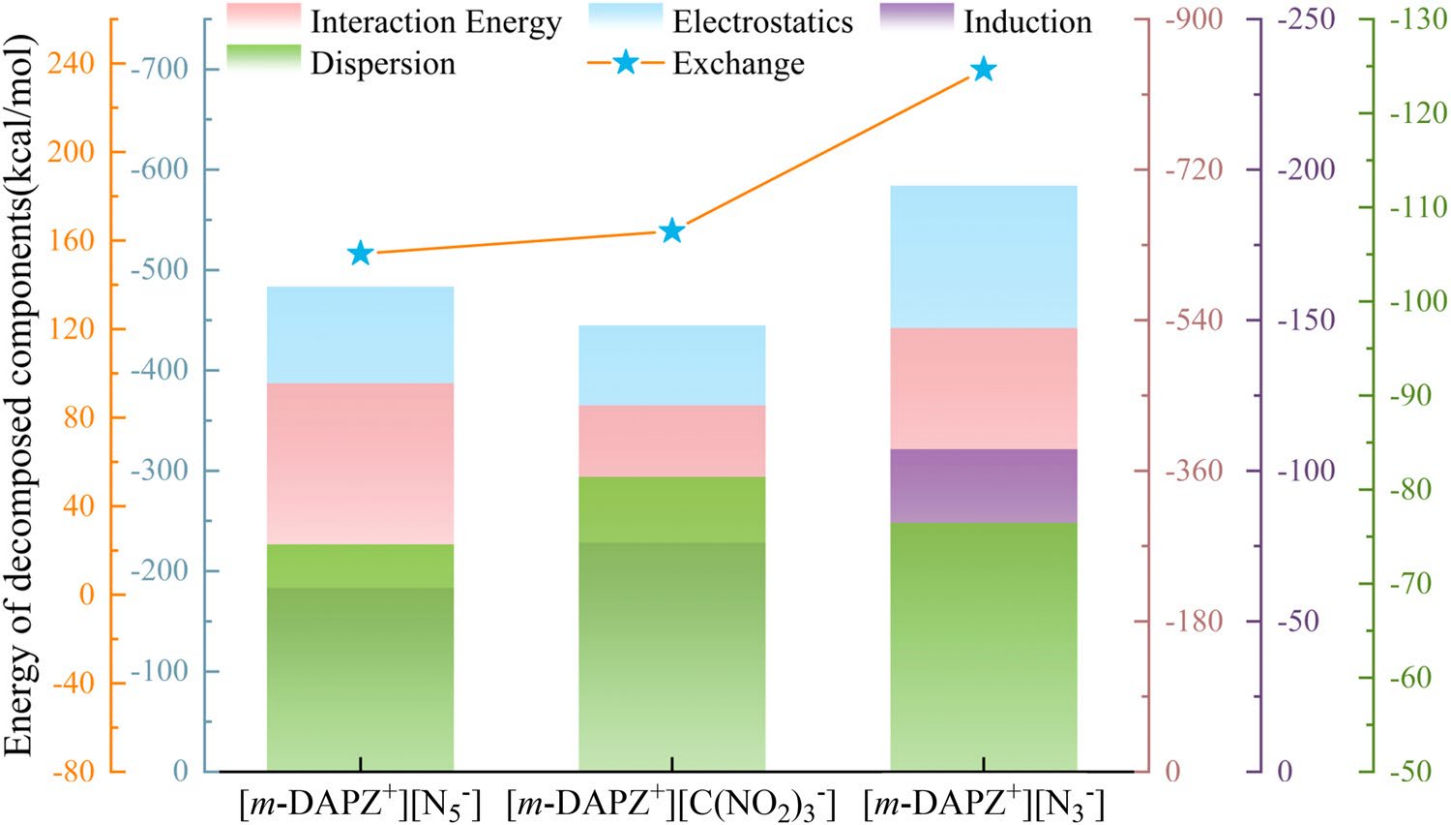
Decomposition diagram of interaction energy of three ions based on SAPT theory.

The interaction energy of the three systems is decomposed and analyzed based on SAPT, as shown in Fig. 8. Generally speaking, among the three systems, electrostatic interaction plays a key role in stabilizing the composite system. Of course, although the contribution of dispersion and induction to the interaction between anions and cations is not as great as that of electrostatic interaction, it cannot be ignored. However, there is a negative correlation between the exchange mutual exclusion energy and the interaction between anion and cation. It is obvious from the figure that although the atom number of N_3_^−^ is the least, its exchange mutual exclusion energy is the highest. This is mainly because the strong polarity of N_3_^−^ has a great negative effect on the electron density between *m-*DAPZ^+^ and N_3_^−^, leading to the weakening of the ion stabilization energy, which reflects the steric effects. Because of its conjugated structure, N_5_^−^ has the lowest polarity, so the repulsion with *m-*DAPZ^+^ is also the lowest. Nevertheless, some new conclusions can be drawn from calculating more accurate data. It has to be mentioned that although there are more hydrogen bonds or larger van der Waals interaction regions in the systems of [*m-*DAPZ^+^][C(NO_2_)_3_^−^] and [*m-*DAPZ^+^][N_5_^−^], in fact, the stronger polarity of N_3_^−^ will also bring some good aspects. From the overall interaction energy, the interaction energy of [*m-*DAPZ^+^][N_3_^−^] system is -530.59 kJ·mol^−1^, which is the smallest among the three systems. It is precisely because of the particularity of N_3_^−^ electron system that electrostatic interaction is greater, and the mutual polarization and mutual transfer of charges between anion and cation are stronger (induced interaction), and their values are −584.11 kJ·mol^−1^ and −107.21 kJ·mol^−1^ respectively. It needs to be explained that this is the biggest as the exchange of mutual exclusion energy, and it is not in conflict. In contrast, the attraction interaction is more dominant. As for the systems of [*m-*DAPZ^+^][C(NO_2_)_3_^−^] and [*m-*DAPZ^+^][N_5_^−^], it is obvious that the electrostatic attraction in the system of [*m-*DAPZ^+^][N_5_^−^] is stronger, which is also determined by the stacking of conjugated *π–π*. Therefore, it is not surprising that the [*m-*DAPZ^+^][C(NO_2_)_3_^−^] ion system has the lowest interaction energy.

### ESP analysis

**Figure 9:**
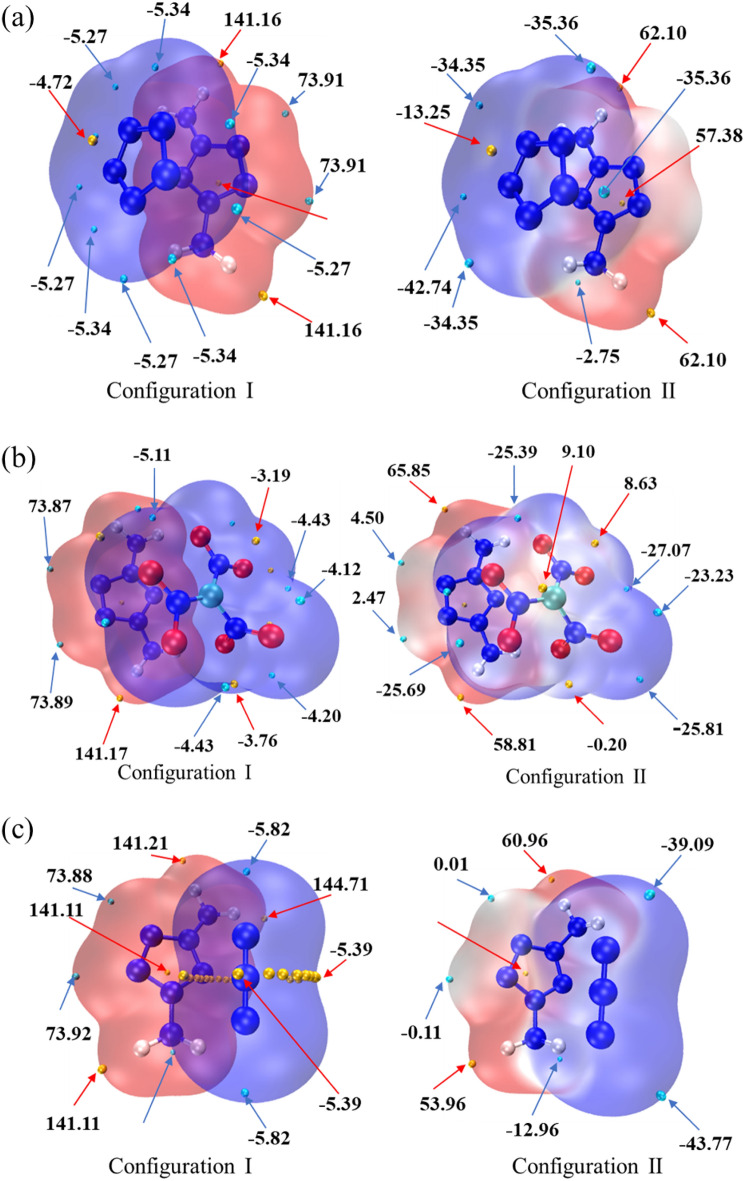
Van der Waals surface diagram and penetration diagram of electrostatic potential of [*m-*DAPZ^+^][N_5_^−^], [*m-*DAPZ^+^][C(NO_2_)_3_^−^], and [*m-*DAPZ^+^][N_3_^−^] (**a–c**).

Electrostatic potential (ESP) is widely used to study the intermolecular electrostatic interaction^38,39^ and predict the reaction sites and molecular properties, so it is necessary to study the electrostatic potential of the system. Generally, the electrostatic potential at point *r* is equal to the electrostatic interaction energy between the unit positive charge placed at that point and the molecule^40,41^. According to Bader's suggestion, electrostatic potential is usually studied on the surface of van der Waals with an electron density of 0.001 a.u.^42,43^ Fig. 9 shows the surface diagrams of van der Waals colored by electrostatic potential of different systems and the surface penetration diagrams of van der Waals between anion and cation. Among them, different colors represent different electrostatic potential values, the redder the color, the more positive the electrostatic potential is, and the bluer the color, the more negative it is. The local minimum and maximum of electrostatic potential on van der Waals surface are drawn as cyan and orange spheres respectively.

According to the principle of electrostatic potential complementarity, the positive region of electrostatic potential is easy to interact with the negative region^44^. It is not difficult to see from Fig. 9 that the positive potential region and negative potential region between *m-*DAPZ^+^ and other anions penetrate each other, which reflects the complementary characteristics of electrostatic potential and also reflects the nature of electrostatic attraction interaction such as hydrogen bond and conjugated *π–π*. At the same time, this means that there is a strong attraction between anion and cation, which helps to form a stable composite system. The electrostatic potential on the upper surface of *m-*DAPZ^+^ is distributed in a wide area, and the maximum and minimum values are about 141.16 kcal·mol^−1^ and 73.90 kcal·mol^−1^ respectively, which are the same in the three systems. In addition, the maximum values are basically near the amino group, which is mainly because the amino group is an electron-donating group, which makes the electrostatic potential on the surrounding surface appear positive. Correspondingly, the minimum values are all around the N atom on the pentazole, which is far away from the amino group and the electron density is relatively high. In the electrostatic potential diagrams of three anion systems, the minimum electrostatic potential is −5.34 kcal·mol^−1^, −5.11 kcal·mol^−1^ and −5.82 kcal·mol^−1^ respectively, and the minimum electrostatic potential of N_3_^−^ anion system is even smaller. In addition, the maximum point of its electrostatic potential is annular near the middle N atom, and its values are all −5.39 kcal·mol^−1^. By comparing the van der Waals surface penetration diagram between ions with the whole van der Waals surface diagram, it can be found that the electron density of the whole ion system has changed greatly after the formation of the composite ion system. The values of extreme points in the positive potential region of *m-*DAPZ^+^ van der Waals surface basically show a decreasing trend, which will mean that the electron density near *m-*DAPZ^+^ increases. Compared with *m-*DAPZ^+^ system alone, the polarity of some of its reaction sites decreases, and *m-*DAPZ^+^ in the composite system will not easily react with external nucleophilic groups. However, the extreme points on the surfaces of the three anions van der Waals also show a decreasing trend, which shows that they are more likely to produce sites that react with external electrophilic groups. Generally speaking, *m-*DAPZ^+^ is assembled with other anions, and they interact with each other and have strong attraction, which helps to stabilize the formation of the complex.

### Energetic performance of assembled ion system

For a brand-new energetic compound with polynitrogen, its energetic performance is an eternal topic. The densities of these ionic compounds were calculated according to the methods proposed by Politzer and Rice (detailed information in SI)^45–47^. The enthalpy of formation^48,49^ of [*m-*DAPZ^+^][N_3_^−^], [*m-*DAPZ^+^][N_5_^−^], and [*m-*DAPZ^+^][C(NO_2_)_3_^−^] was estimated to be 1113.14 kJ·mol^−1^, 1042.03 kJ·mol^−1^, and 208.09 kJ·mol^−1^. Finally, according to the K-J equation proposed by Kamlet and Jacobs^50,51^, the detonation pressures and detonation velocities of the three systems were calculated. In addition, EXPLO5 software^52^ was used to calculate the energetic properties to supplement it.

The data of density, detonation velocity and detonation pressure are summarized in Table 2, from which we can get very exciting information. The theoretical detonation velocities of the assembled three ion systems are all above 8500 m·s^−1^ or 9000 m·s^−1^, which are calculated respectively by K–J equation or EXPLO5 V6.05. In addition, the detonation velocity of [*m-*DAPZ^+^][N_5_^−^] is even close to CL-20 (the calculation result of EXPLO5 exceeds 10,000 m·s^−1^). While [*m-*DAPZ^+^][N_3_^−^] will hopefully replace conventional explosives because of its super high specific impulse (319.8 s), and then play a great role in the aerospace and some areas with specific needs. Finally, comparing the detonation performance of [*m-*DAPZ^+^][C(NO_2_)_3_^−^] with the well-known oxidizer [N_2_H_5_^+^] [C(NO_2_)_3_^−^], it is not difficult to find that their oxygen balance and energy are comparable. The results show that [*m-*DAPZ^+^][C(NO_2_)_3_^−^] is a new high energetic oxidizer which is very suitable to form a mixed system with negative oxygen balance explosives. What needs to be added is that in the calculation process, even though experts and scholars have supplemented some theoretical formulas in detail, in fact, these formulas still have some errors due to the limitations of the theory and the complexity of the composite system. Especially, when calculating the density, although Rice and others repeatedly consider the weak interaction between anion and cation, it is not difficult to see from the original text that the interaction of LP–*π*, *π–π*, etc. are not considered. It will mean that the volume calculated is still too large, which will lead to the decrease of the calculated density. On the contrary, its theoretical density will be greater, and of course, its energy will increase, the importance of its research is self-evident. Therefore, the appearance of the above three high energetic materials, especially [*m-*DAPZ^+^][N_5_^−^], will be very exciting.

**Table 2:** Detonation performance of three systems and other energetic materials.

	*ρ* (g·cm^−3^)^a^	*D* (m·s^−1^)^b^	*P* (GPa)^c^	*I*sp (s)^d^	Δ*H*_f_ (kJ·mol^−1^)^e^	OB (%)^f^
[*m-*DAPZ^+^][N_5_^−^]	1.6487	9246/10,016	35.92/37.94	304.4	1113.14	−18.6
[*m-*DAPZ^+^][N_3_^−^]	1.5373	9170/9765	33.76/35.08	319.8	1042.03	−22.2
[*m-*DAPZ^+^][C(NO_2_)_3_^−^]	1.8624	8508/9062	32.76/32.81	241.4	208.09	+ 12.7
CL-20^g^	2.038	9634/9773	44.21/44.85	272.1	397.80	−11.0
HMX^g^	1.905	9137/9193	38.29/37.85	265.6	74.80	−21.6
HNF^h^	1.86	8922/8948	36.00/34.40	254.4	−72	+ 13.1

^a^Calculated density.

^b^Detonation velocity, values calculated using the K–J equation/EXPLO5 V6.05.

^c^Detonation pressure, values calculated using the K–J equation/EXPLO5 V6.05.

^d^Specific impulse of the neat compound using the EXPLO5 V6.05 program package at 7 Mbar chamber pressure.

^e^Calculated heat of formation.

^f^Oxygen balance (based on CO_2_) for C_a_H_b_O_c_N_d_, 1600(c − 2a − b/2)/M_W_; M_W_ is the  molecular weight.

^g^Ref.^53^.

^h^HNF: [N_2_H_5_^+^][C(NO_2_)_3_^−^], Ref.^54^.

## Conclusion

In this study, DAPZ^+^ and its assembled polynitrogen and high-energy ionic compounds were studied deeply and comprehensively by combining experimental means with theoretical calculation. Mass spectrometry analysis indicated that diamino-pentazole was first successfully prepared during the amination of [Na(H_2_O)(N_5_)] 2H_2_O by HOSA. The calculation of ground state energy and transition state shows that the reaction product is *m*-DAPZ^+^ in solvent, which is a great breakthrough in the synthesis of pentazole derivatives.

The outstanding performance of DAPZ^+^ is perfectly reflected in the calculation of the assembled system. The results of AIM, IGMH analysis show that DAPZ^+^ and N_5_^−^, N_3_^−^, C(NO_2_)_3_^−^ all have strong interactions, including hydrogen bonds, LP–*π*, *π–π* and extensive van der Waals interactions, which play a positive role in the formation of single crystals. In addition, SAPT results verify the above analysis and calculate the accurate interaction energy. As for the detonation performance of the three systems, the theoretical calculation results are exciting. The theoretical detonation velocities of the three systems are all above 9000 m·s^−1^, especially [*m-*DAPZ^+^][N_5_^−^] reached 10,016 m·s^−1^ (EXPLO5 V6.05 value). Due to the limitations of theoretical methods, their actual detonation velocities will be higher. The specific impulse of [*m-*DAPZ^+^][N_3_^−^] can reach 319.8 s at a relatively low density. Even for [*m-*DAPZ^+^][C(NO_2_)_3_^−^], it is a high-energy oxidizer that can be compared to HNF. These excellent properties may allow the above three kinds of polyanion systems to replace many energetic materials with poor performance at present and play a greater role in military, aerospace and other fields. Therefore, it has to be said that the synthesis of DAPZ^+^ and the exploration of nitrogen-rich and high-energy compounds are relatively successful, which may promote the field of energetic materials to move towards polynitrogen.

## Methods

### Experimental preparation

All reagents and solvents were purchased from Aladdin and Energy Chemical as analytical grade and used without further purification. All experiments used deionized water with a resistivity of 18.2 MΩ.

For the cleavage experiments, these pentazoles reaction liquid were dissolved in CH_3_OH, and infused into the spectrometer’s ion source at 10 μL·min^−1^ with a syringe pump. In tandem mass spectrometry (MS/MS) experiments, the desired negative-ion or positive-ion peaks are mass-selected and subjected to product ion mass analysis following collision-induced dissociation (CID) at variable collision voltages using N_2_ as the collision gas.

Mass spectrometric ionization was carried out in electrospray ionization source (ESI) ionization mode with a source temperature of 250 °C. The detection was carried out on a TSQ Vantage triple quadrupole (QqQ) mass spectrometer (Thermo Fisher Scientific), equipped with an ESI turbo spray ionization source. The entire system was controlled via Xcalibur 2.2 software and data were processed using TraceFinder 3.1.

### Calculation details

In order to further obtain the synthetic route of *m*-DAPZ^+^, the amination process of pentazolate anion by hydroxylamine *O*-sulfonic acid (HOSA) was calculated and analyzed, and the whole process was carried out under Gaussian09 software package^55^. Due to HOSA is easily hydrolyzed to form OSA^-^ anion in solution, the initial amination mechanism of OSA^−^ and N_5_^−^ was calculated at the level of B3LYP-D3/6-311+G** based on the implicit solvation model^56,57^. It should be emphasized that the diffuse function is very important in this work, mainly because the anion system has a significant negative charge and its electrons are easily polarized. Then, on the basis of amino pentazole, the process of amination of OSA^−^ anion to its ortho and meta positions was further explored. In order to make the energy obtained in the whole calculation process of transition state more accurate, the method of DLPNO-CCSD(T)/CBS (ma-def2-TZVPP → QZVPP extrapolation) with tightPNO and RIJK is adopted to calculate the energy of the final product and transition state, etc^58–61^. Among them, the Resolution of Identity (RI) means will be applied to both Coulomb Integrals and HF Exchange Integrals, and the DLPNO threshold is selected as "tight", so the calculation process will be greatly accelerated on the premise of ensuring accuracy^62^. As a supplementary note, in fact, the energy accuracy of this method and basis set is very close to CCSD(T)/CBS, so the calculated energy is very accurate and reliable^63^. Of course, the energy calculation work is carried out under the ORCA5.0.4 software package^64^. And the *m*-DAPZ^+^ was characterized by theoretical calculation. In addition, concerning that *m-*DAPZ^+^ has high energy and potential application value, azide anion ([N_3_^−^]), pentazolate anion ([N_5_^−^]) and nitroform anion ([C(NO_2_)_3_^−^]) are selected to assemble with it, thus forming three high-energy ionic salts. In order to obtain the most stable structure, thirty potential configurations of *m-*DAPZ^+^ and each anion were found at B3LYP-D3/6-311 +G**^65^. After that, five stable configurations were selected from each system to further calculate the electron energy at M062X-D3/ma-def2-TZVPP level^60,61,66^, and finally the most stable structure was selected for subsequent analysis. Interestingly, there is no version of def2 series basis set with diffuse function, so the data of ma-def2-TZVPP basis set is provided additionally^67^. Subsequently, the AIM, ESP, IGMH and detonation parameters of the three selected systems ([*m-*DAPZ^+^][N_5_^−^], [*m-*DAPZ^+^][N_3_^−^], [*m-*DAPZ^+^][C(NO_2_)_3_^−^]) were calculated. All thermodynamic quantities of ionic systems are calculated by thermodynamic combination method G4(MP2)-6X^68^. Especially, according to the interaction energy of anion and cation in the three systems, the energy decomposition calculation was carried out with the "gold standard" SAPT2+(3)dMP2/aug-cc-pVTZ under PSI4 software package^69,70^, which is based on Symmetry-Adapted Perturbation Theory (SAPT)^71^. Finally, it is worth noting that part of the analysis process will be carried out under Multiwfn software^72^.

### Supplementary Information

Supplementary Video 1.

Supplementary Video 2.

Supplementary Video 3.

Supplementary Video 4.

Supplementary Information.

## Data Availability

All data generated and analyzed during this study are included in this article, its Supplementary Information, and also available from the authors upon reasonable request.

## References

[1] Cacace F, Patris G, Troiani A (2002). Experimental detection of tetranitrogen. Science.

[2] Christe KO, Wilson WW, Sheehy JA, Boatz JA (1999). N_5_^+^: A novel homoleptic polynitrogen ion as a high energy density material. Angew. Chem. Int. Ed..

[3] Eremets MI, Gavriliuk AG, Trojan IA, Dzivenko DA, Boehler R (2004). Single-bonded cubic form of nitrogen. Nat. Mater..

[4] Christe KO (2007). Recent advances in the chemistry of N5^+^, N5^−^ and high-oxygen compounds. Propellants Explos. Pyrotech..

[5] Zarko VE (2010). Searching for ways to create energetic materials based on polynitrogen compounds (review). Combust. Explos. Shock Waves.

[6] Xu YG (2017). A series of energetic metal pentazolate hydrates. Nature.

[7] Zhang C, Sun CG, Hu BC, Yu CM, Lu M (2017). Synthesis and characterization of the pentazolate anion *cyclo*-N_5_ˉ in (N_5_)_6_(H_3_O)_3_(NH_4_)_4_Cl. Science.

[8] Zhang WQ (2018). Stabilization of the pentazolate anion in a zeolitic architecture with Na_20_N_60_ and Na_24_N_60_ nanocages. Angew. Chem. Int. Ed..

[9] Wang PC, Xu YG, Lin QH, Lu M (2018). Recent advances in the syntheses and properties of polynitrogen pentazolate anion *cyclo*-N_5_^−^ and its derivatives. Chem. Soc. Rev..

[10] Wozniak DR, Piercey DG (2020). Review of the current synthesis and properties of energetic pentazolate and derivatives. Thereof. Eng..

[11] Luo J, Xia H, Zhang W, Song S, Zhang Q (2020). A promising hydrogen peroxide adduct of ammonium cyclopentazolate as a green propellant component. J. Mater. Chem. A.

[12] Zhou, J. *et al*. Improving the stability of hydrazinium pentazolate through cocrystallization. *CrystEngComm* **25** (2023).

[13] Huisgen R, Ugi I (1956). Zur Lösung eines klassischen problems der organischen stickstoff-chemie. Angew. Chem. Int. Ed..

[14] Ugi I, Huisgen R, Clusius K, Vecchi M (1956). Zur reaktion des benzol-diazonium-ions rnit azid nachweis des phenyl-pentazols als zwischenstufe. Angew. Chem. Int. Ed..

[15] Huisgen R, Ugi I, Pentazole I (1957). Die Lösung eines klassischen problems der organischen stickstoffchemie. Chem. Ber..

[16] Ugi I, Huisgen R (1958). Die zerfallsgeschwindigkeit der aryl-pentazole. Chem. Ber..

[17] Ugi I, Perlinger H, Behringer L (1958). Kristallisierte aryl-pentazole. Chem. Ber..

[18] Wallis JD, Dunitz JD (1983). An all-nitrogen aromatic ring system: Structural study of 4-dimethyl-aminophenylpentazole. J. Chem. Soc. Chem. Commun..

[19] Schroer T, Haiges R, Schneider S, Christe KO (2005). The race for the first generation of the pentazolate anion in solution is far from over. Chem. Commun..

[20] Butler RN, Stephens JC, Hanniffy JM (2004). First reversible protonation of the all-nitrogen 1-aryl pentazole ring. Tetrahedron Lett..

[21] Benin V, Kaszynski P, Radziszewski JG (2002). Arylpentazoles revisited: experimental and theoretical studies of 4-hydroxyphenylpentazole and 4-oxophenylpentazole anion. J. Org. Chem..

[22] Östmark H (2003). Detection of pentazolate anion (*cyclo*-N_5_^−^) from laser ionization and decomposition of solid *p*-dimethylaminophenylpentazole. Chem. Phys. Lett..

[23] Bi Y (2010). Three *p*-*tert*-butylthiacalix [4] arene-supported cobalt compounds obtained in one pot involving in situ formation of N_6_H_2_ ligand. Inorg. Chem..

[24] Banert K, Pester T (2020). Nucleophilic attack of azide at electrophilic azides: Formation of N_6_ units in hexazene and aminopentazole derivatives. Angew. Chem. Int. Ed..

[25] Xu Z (2023). Why does the cyclic pentazolate anion fail to undergo N-oxidization in oxone solution?. New J. Chem..

[26] Bondarchuk SV (2021). Structure enhancement of energetic materials: A theoretical study of the arylamines to arylpentazoles transformation. FirePhysChem.

[27] Bader RFW (2002). A quantum theory of molecular structure and its applications. Chem. Rev..

[28] Sholl, D. S. & Steckel, J. A. *Density Functional Theory—A Practical Introduction* (ed. Sholl, D. S.). 13–15 (Wiley, 2009).

[29] Bader RFW, Beddall PM (1972). Virial field relationship for molecular charge distributions and the spatial partitioning of molecular properties. J. Chem. Phys..

[30] Humphrey W, Dalke A, Schulten K (1996). VMD: Visual molecular dynamics. J. Mol. Graph. Model..

[31] Espinosa E, Alkorta I, Elguero J, Molins E (2002). From weak to strong interactions: A comprehensive analysis of the topological and energetic properties of the electron density distribution involving X-H⋯F–Y systems. J. Chem. Phys..

[32] Koch U, Popelier PLA (2002). Characterization of C–H–O hydrogen bonds on the basis of the charge density. J. Phys. Chem..

[33] Rozas I, Alkorta I, Elguero J (2000). Behavior of ylides containing N, O, and C atoms as hydrogen bond acceptors. J. Am. Chem. Soc..

[34] Johnson ER (2010). Revealing noncovalent interactions. J. Am. Chem. Soc..

[35] Lefebvre C (2017). Accurately extracting the signature of intermolecular interactions present in the NCI plot of the reduced density gradient versus electron density. Phys. Chem. Chem. Phys..

[36] Lu T, Chen Q (2022). Independent gradient model based on Hirshfeld partition: A new method for visual study of interactions in chemical systems. J. Comput. Chem..

[37] Mooibroek TJ, Gamez P, Reedijk J (2008). Lone pair–*π* interactions: A new supramolecular bond?. CrystEngComm.

[38] Sun KB, Zhang SH, Ren FD, Hao YP, Ba SH (2021). Theoretical prediction of the trigger linkage, cage strain, and explosive sensitivity of CL-20 in the external electric fields. J. Mol. Model..

[39] Liu ZY, Lu T, Chen QX (2021). Intermolecular interaction characteristics of the all-carboatomic ring, cyclo[18]carbon: Focusing on molecular adsorption and stacking. Carbon.

[40] Lu T, Manzetti S (2014). Wavefunction and reactivity study of benzo [a] pyrene diol epoxide and its enantiomeric forms. Struct. Chem..

[41] Rice BM, Hare JJ (2002). A quantum mechanical investigation of the relation between impact sensitivity and the charge distribution in energetic molecules. J. Phys. Chem. A.

[42] Bader RFW, Carroll MT, Cheeseman JR, Chang C (2002). Properties of atoms in molecules: Atomic volumes. J. Am. Chem. Soc..

[43] Murray JS, Politzer P (2011). The electrostatic potential: An overview. Wiley Interdiscip. Rev. Comput. Mol. Sci..

[44] Lu T, Chen F (2013). Revealing the nature of intermolecular interaction and configurational preference of the nonpolar molecular dimers (H_2_)_2_, (N_2_)_2_, and (H_2_)(N_2_). J. Mol. Model..

[45] Politzer P, Martinez J, Murray JS, Concha MC, ToroLabbe A (2009). An electrostatic interaction correction for improved crystal density prediction. Mol. Phys..

[46] Politzer P, Martinez J, Murray JS, Concha MC (2010). An electrostatic correction for improved crystal density predictions of energetic ionic compounds. Mol. Phys..

[47] Rice BM, Hare JJ, Byrd EFC (2007). Accurate predictions of crystal densities using quantum mechanical molecular volumes. J. Phys. Chem. A.

[48] Jenkins HDB, Roobottom HK, Passmore J (1999). Relationships among ionic lattice energies, molecular (formula unit) volumes, and thermochemical radii. Inorg. Chem..

[49] Jenkins HDB, Tudela D, Glasser L (2002). Lattice potential energy estimation for complex ionic salts from density measurements. Inorg. Chem..

[50] Kamlet MJ, Jacobs SJ (1968). Chemistry of detonations I A simple method for calculating detonation properties of C-H–N–O explosives. J. Chem. Phys..

[51] Dong K (2013). Formyl azido substituted nitro hexaazaisowurtzitane—Synthesis, characterization and energetic properties. New J. Chem..

[52] Sućeska, M. *EXPLO5 V6.05.04*. (Brodarski Institute, 2020).

[53] Keshavarz MH, Abadi YH (2018). Novel organic compounds containing nitramine groups suitable as high-energy cyclic nitramine compounds. ChemistrySelect.

[54] Yu Q (2021). Bridged and fused triazolic energetic frameworks with an azo building block towards thermally stable and applicable propellant ingredients. J. Mater. Chem. A.

[55] Frisch, M. J. *et al*. *Revision E.01*. (Gaussian Inc., 2013).

[56] Becke AD (1988). Density-functional exchange-energy approximation with correct asymptotic behavior. Phys. Rev. A.

[57] Becke AD, Johnson ER (2005). A density-functional model of the dispersion interaction. J. Chem. Phys..

[58] Liakos DG, Neese F (2012). Improved correlation energy extrapolation schemes based on local pair natural orbital methods. J. Phys. Chem. A.

[59] Neese F, Valeev EF (2011). Revisiting the atomic natural orbital approach for basis sets: Robust systematic basis sets for explicitly correlated and conventional correlated Ab initio methods?. J. Chem. Theory Comput..

[60] Weigend F, Ahlrichs R (2005). Balanced basis sets of split valence, triple zeta valence and quadruple zeta valence quality for H to Rn: Design and assessment of ac- curacy. Phys. Chem. Chem. Phys..

[61] Zheng J, Xu X, Truhlar DG (2011). Minimally augmented Karlsruhe basis sets. Theor. Chem. Acc..

[62] Weigend F (2002). A fully direct RI-HF algorithm: Implementation, optimised auxiliary basis sets, demonstration of accuracy and efficiency. Phys. Chem. Chem. Phys..

[63] Goerigk L, Grimme S (2011). A thorough benchmark of density functional methods for general main group thermochemistry, kinetics, and noncovalent interactions. Phys. Chem. Chem. Phys..

[64] Neese F, Wennmohs F, Becker U, Riplinger C (2020). The ORCA quantum chemistry program package. J. Chem. Phys..

[65] Lu, T. *Molclus Program Rev. 1.9.9*. http://www.keinsci.com/research/molclus.html (2020).

[66] Zhao Y, Truhlar DG (2007). The M06 suite of density functionals for main group thermochemistry, thermochemical kinetics, noncovalent interactions, excited states, and transition elements: Two new functionals and systematic testing of four M06-class functionals and 12 other functionals. Theor. Chem. Acc..

[67] Papajak E, Zheng J, Xu X, Leverentz HR, Truhlar DG (2011). Perspectives on basis sets beautiful: Seasonal plantings of diffuse basis functions. J. Chem. Theory Comput..

[68] Chan B, Deng J, Radom L (2011). G4 (MP2)-6X: A cost-effective improvement to G4 (MP2). J. Chem. Theory Comput..

[69] Parker TM, Burns LA, Parrish RM, Ryno AG, Sherrill CD (2014). Levels of symmetry adapted perturbation theory (SAPT). I. Efficiency and performance for interaction energies. J. Chem. Phys..

[70] Patkowski K (2020). Recent developments in symmetry-adapted perturbation theory. Wiley Interdiscip. Rev. Comput. Mol. Sci..

[71] Szalewicz K (2012). Symmetry-adapted perturbation theory of intermolecular forces. Wiley Interdiscip. Rev. Comput. Mol. Sci..

[72] Lu T, Chen F (2012). Multiwfn: A multifunctional wavefunction analyzer. J. Comput. Chem..

