# Supplementary material for: Experimental detection of the diamino-pentazolium cation and theoretical exploration of derived high energy materials
Source: Sci Rep. 2024 May 2;14:10120. doi: 10.1038/s41598-024-60741-z (PMC11065884; doi:10.1038/s41598-024-60741-z)
Supplement: Supplementary file 5 — Supplementary Information. [file 41598_2024_60741_MOESM5_ESM.docx]

**Supplementary Information**

**Experimental detection of the diamino-pentazolium cation and theoretical exploration of derived high energy materials**

**Tianyang Hou^1^, Xiaofeng Yuan^1^, Shuaijie Jiang, Ze Xu, Xiaopeng Zhang, Ming Lu* and Yuangang Xu***

**School of Chemistry and Chemical Engineering, Nanjing University of Science and Technology, Nanjing 210094, China**

**E-mail: luming@njust.edu.cn (M.L.); yuangangxu@163.com (Y.X.)**

**^1^These two authors contributed equally.**

| **Table of Contents** | | |
| --- | --- | --- |
| 1. | Experimental section | S2 |
| 2. | Computational Details | S4 |
| 3. | Key Geometric Features of ***m*-2** | S9 |
|  | References | S11 |

1. **Experimental section**

1.1 Caution

Although no explosion or hazards were observed during the preparation and handling of these energetic compounds, all the materials investigated are potentially energetic materials. Small-scale syntheses are strongly encouraged. Manipulations must be carried out in a hood behind a safety shield. Eye protection and leather gloves must be worn at all times.

1.2 Synthesis procedures

NaN_5_ was synthesized according to our previously published procedures.^1^

0.72 g sodium hydroxide (18 mmol) and 2.3 g potassium dihydrogen phosphate (17 mmol) were dissolved in 10 mL of distilled water at room temperature, there is an exothermic phenomenon in the process of dissolution. After the solution was cooled to room temperature, 0.147 g of Na(H_2_O)(N_5_)∙2H_2_O (1 mmol) solution (10 mL of water) was added and stirred for 10 minutes. 0.45 g (4 mmol) of hydroxylamine-O-sulfonic acid (HOSA) was added to the reaction solution and stirred vigorously. After the reaction at 40 ^o^C for 8 hours, the sample was taken for mass spectrometry test.

1.3 Mass spectra

The reaction solution was diluted and filtered with chromatographic grade methanol, and infused into the spectrometer’s ion source at 30 mL min^-1^ with a syringe pump. In tandem mass spectrometry (MS-MS) experiments, the desired negative-ion peaks were mass-selected and subjected to product ion mass analysis following collision-induced dissociation (CID) at variable collision voltages using N_2_ or Ar as the collision gas.


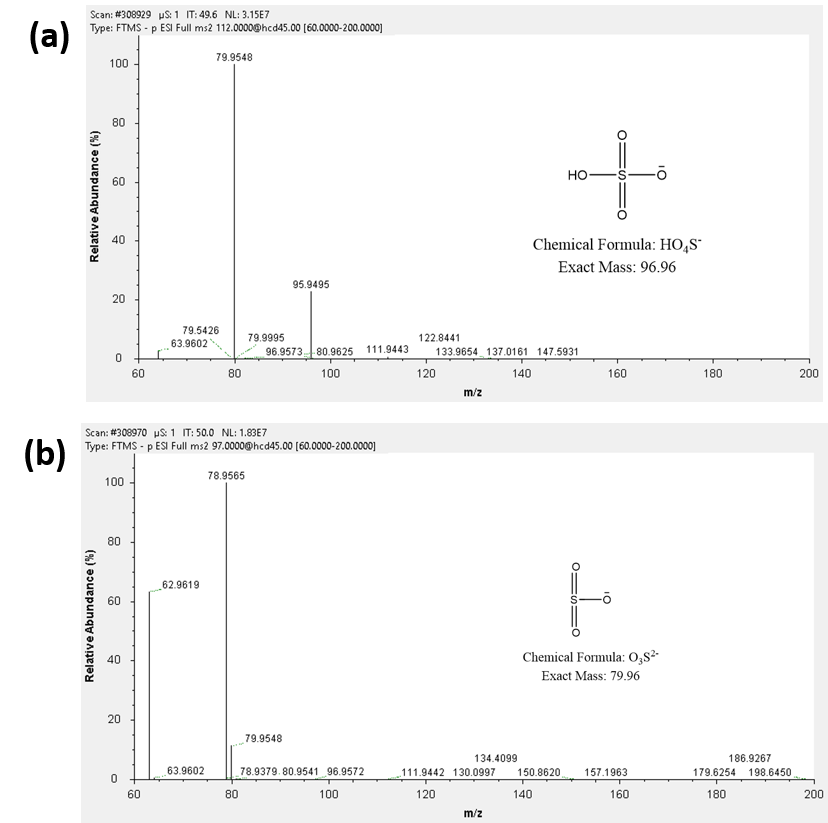


**Fig. S1.** Negative ion, full-range CID mass spectra of the mass selected (m/z 97) and (m/z 79) peaks

1. **Computational Details**

The molecular optimization and frequency calculations were performed by the *Gaussian 09* package^2^ under the G4MP2 method. All of the optimized structures were characterized to be a true local energy minimum on potential energy surfaces without imaginary frequencies. Based on the optimized structures, atomic dipole moment corrected Hirshfeld (ADCH), the surface electrostatic potential (ESP), highest occupied molecular orbital (HOMO), lowest unoccupied molecular orbital (LUMO), infrared (IR) and NMR spectra, pathway of *π* electron delocalization and the *π* electron density distribution diagram were obtained.


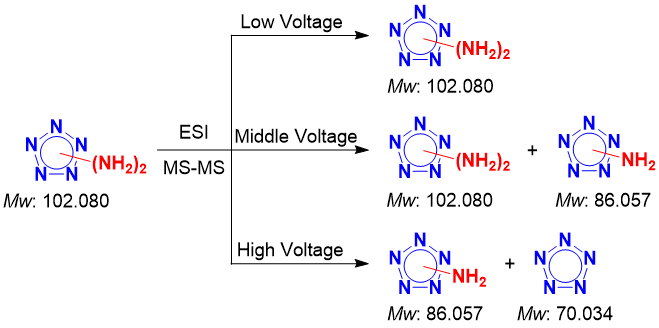


**Fig. S2.** ESI-MS-MS fragmentations of the diamino-substituted pentazole species at low, middle, and high collision voltages.


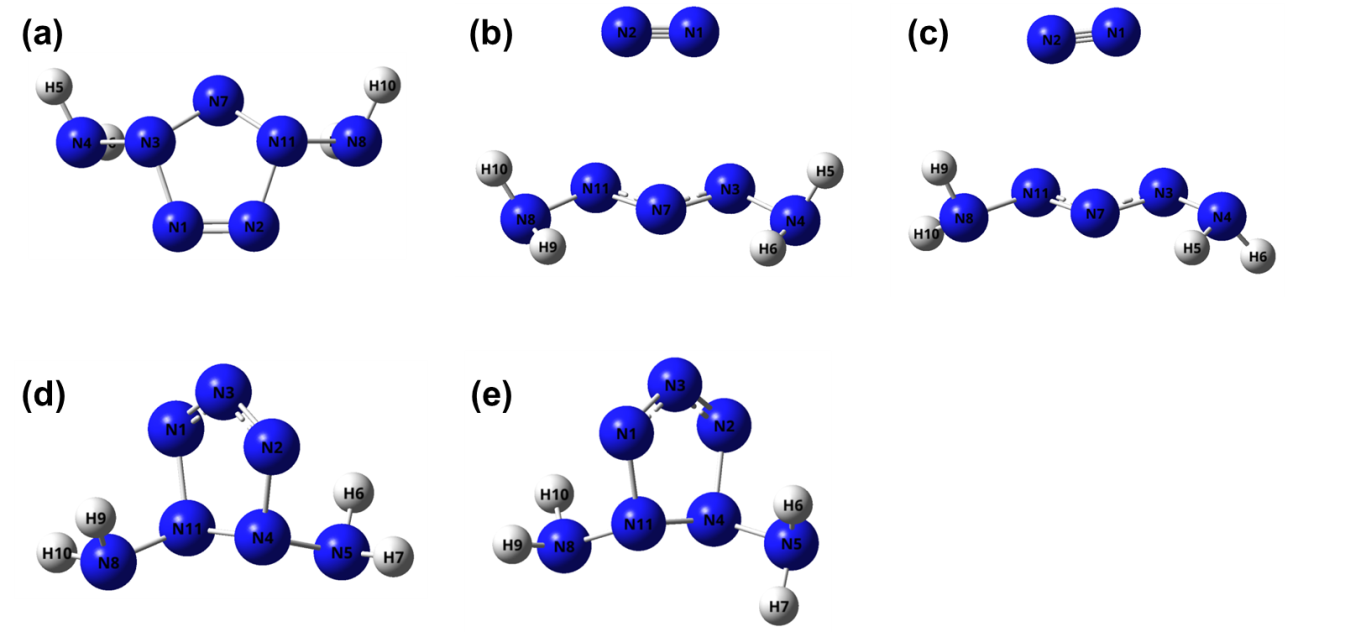


**Fig. S3.** Unreasonable Optimized structures of the H_4_N_7_ anion.


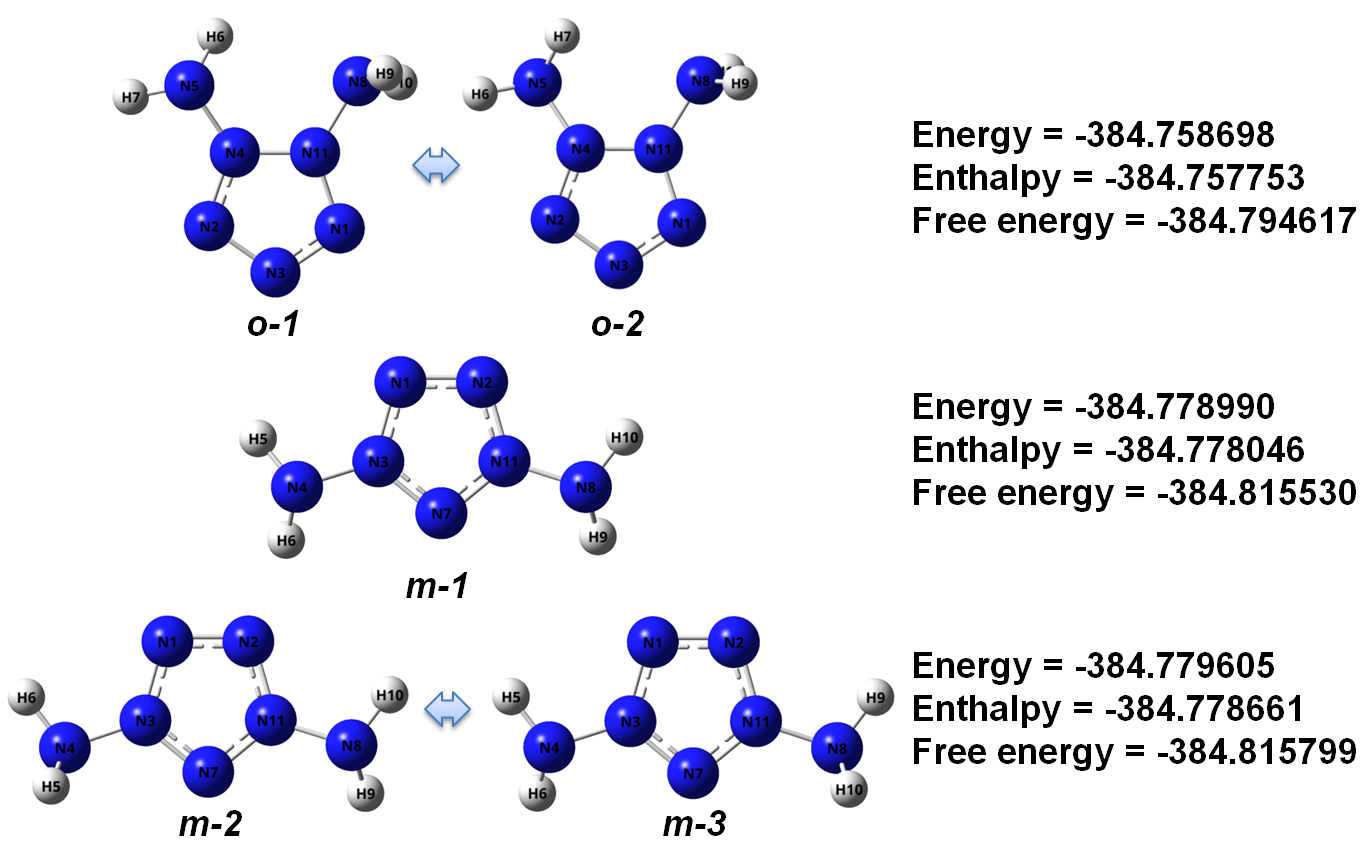


**Fig. S4.** Optimized structures of the **DAPZ^+^** isomers (angstroms) and their energies (in a.u.) calculated at the M06-2X/6-31+G(d,p) level.


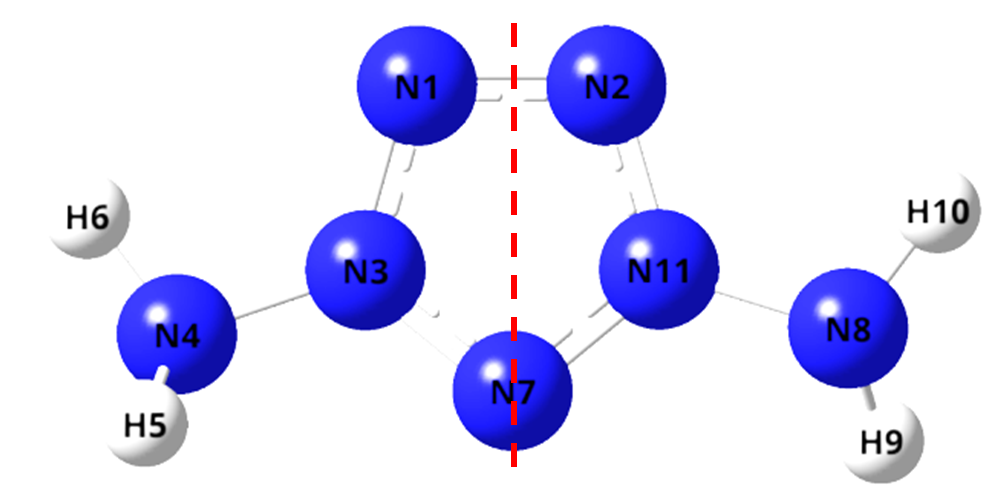


**Fig. S5.** Structure of ***m*-2** with atomic labelling and *C*_2_ axis.


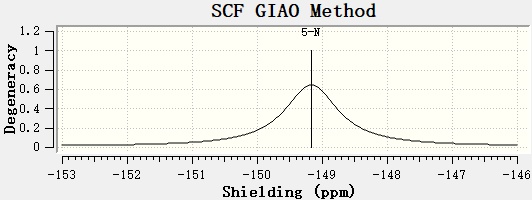


**Fig. S6.** ^15^N NMR prediction of nitro-methane as TMS.

**
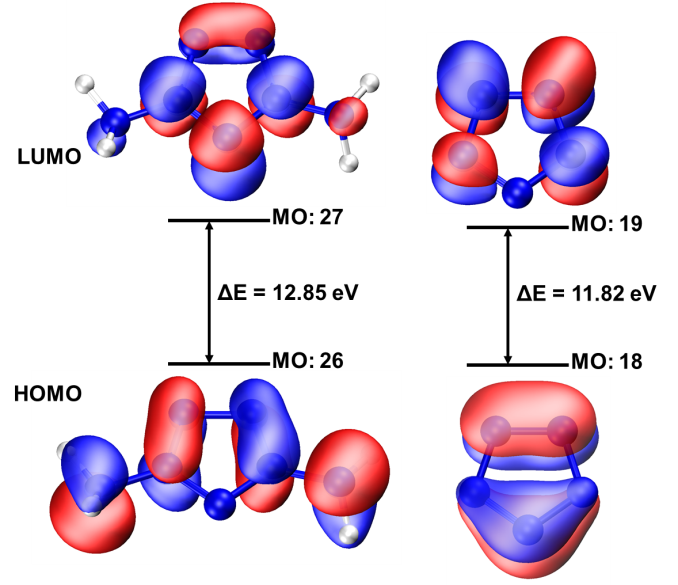
**

**Fig. S7.** HOMO and LUMO energy levels and energy gaps of ***m*-2**.

The density of ionic compounds should be calculated first, and the calculation formula is shown equations (1) to (3).^3-5^

$\text{V}_{\text{uncorrected}}\text{=}\text{p}\text{V}_{\text{M}^{\text{+}}}\text{+}\text{q}\text{V}_{\text{X}^{\text{-}}}$ (1)

$\text{V}_{\text{corrected}}\text{=}\text{V}_{\text{uncorrected}}\text{-[0.6763+0.9418}\text{N}\text{]}$ (2)

$\text{ρ}\left( \text{crystal} \right)\text{=}\text{α}\text{(M/}\text{V}_{\text{corrected}}\text{)+}\text{β}\text{(}\text{V}_{\text{s}^{\text{+}}}\text{/}\text{A}_{\text{s}^{\text{+}}}\text{)+}\text{γ}\text{(}\text{V}_{\text{s}^{\text{-}}}\text{/}\text{A}_{\text{s}^{\text{-}}}\text{)+δ}$ (3)

For the ionic compound *M_p_X_q_*, its volume is not as in equation (1), simply the sum of anion and cation. Rice modified it according to the weak interaction between ionic systems. 0.6763 and 0.9418 are fitting parameters, and *N* represents the number of hydrogen bonds. Then, according to the density formula of energetic ion salt proposed by Politzera, the theoretical density can be obtained, where M is the molar mass of the salt, $\text{V}_{\text{s}^{\text{+}}}$, $\text{V}_{\text{s}^{\text{-}}}$ are the average electrostatic potentials of anion and cation, *s*^+^, *s*^-^ is the surface area of positive electrostatic potential cation, and the values of *α*, *β*, *γ* and δ are 1.0260, 0.0514, 0.0419 and 0.0227.

As for the enthalpy of formation, although atomization method plays an important role in solving the enthalpy of formation of covalent compounds, it is obvious that the prediction method of enthalpy of formation proposed by Jenkins is more suitable for energetic ionic compounds, such as equations (4) to (6).^6,7^

${\text{∆}\text{H}}_{\text{f}}\left( \text{salt}\text{, 298 K} \right)\text{=∆}\text{H}_{\text{f}}\left( \text{anion}\text{, 298K} \right)\text{+∆}\text{H}_{\text{f}}\left( \text{anion}\text{, 298K} \right)\text{-∆}\text{H}_{\text{L}}$ (4)

$\text{∆}\text{H}_{\text{L}}\text{=}\text{U}_{\text{pot}}\text{+[}\text{p}\text{(}\text{n}_{\text{M}}\text{/2-2)+}\text{q}\text{(}\text{n}_{\text{X}}\text{/2-2)]}\text{RT}$ (5)

$\text{U}_{\text{POT}}\left[ \text{kJ}{\cdot\text{mol}}^{\text{-1}} \right]\text{=}\text{γ}{\text{(}\text{ρ}_{\text{m}}\text{/}\text{M}_{\text{m}}\text{)}}^{\text{1/3}}\text{+}\text{δ}$ (6)

Jenkins thinks that lattice energy $\text{U}_{\text{POT}}$ plays an important role in predicting the enthalpy of formation of ionic compounds. Therefore, after calculating the sum of the formation enthalpies of anion and cation, it is necessary to consider the lattice enthalpy $\text{H}_{\text{L}}$ additionally. In the formula, *ρ*_m_ is the density of salt, $\text{M}_{\text{m}}$ is the mass of chemical ionic materials, and *γ* and *δ* are the empirical values of Jenkins fitting.

Finally, according to the K-J equation proposed by Kamlet and Jacobs,^8^ the detonation pressures and velocities of the three systems are calculated, as shown in equations (7) to (8).

$\text{D}\text{=1.01}{{\text{(N}\bar{\text{M}}}^{\text{1/2}}\text{Q}^{\text{1/2}}\text{)}}^{\text{1/2}}\text{(1+1.3}\text{ρ}\text{)}$ (7)

$\text{P}\text{=1.558}\text{ρ}^{\text{2}}\text{N}{\bar{\text{M}}}^{\text{1/2}}\text{Q}^{\text{1/2}}$ (8)

Where *Q* is the heat of explosion, N is the number of moles, the average molecular weight of m produced per gram of gas is the gaseous product, and *ρ* is the crystal density. It should be noted that for *m-*DAPZ^+^ system, which contains four elements of C*_a_*H*_b_*O*_c_*N*_d_*, the relationship between the explosion heat and the enthalpy of formation is shown in equation (9). But there are only N and H atoms in System 1 and 2, their relationship between explosion heat and enthalpy of formation is shown in equation (10).^9^

$\text{Q}\text{∙}\text{10}^{\text{-3}}\text{=(28.9b+94.05a+0.239∆}\text{H}_{\text{f}}\text{)/}\text{M}$ (9)

$\text{Q}\text{∙}\text{10}^{\text{-3}}\text{=(57.8c+0.239∆}\text{H}_{\text{f}}\text{)/}\text{M}$ (10)

1. **Key Geometric Features of *m*-2**

**Table S1.** N–N Bond lengths (Å) of ***m*-2**

| **N1-N2** | 1.305 |
| --- | --- |
| **N2-11** | 1.322 |
| **N11-N7** | 1.314 |
| **N7-N3** | 1.314 |
| **N3-N1** | 1.322 |
| **N3-N4** | 1.359 |
| **N11-N8** | 1.359 |

**Table S2.** Bond angles of ***m*-2** (^o^)

| **N3-N1-N2** | 105.660 |
| --- | --- |
| **N1-N2-N11** | 105.661 |
| **N2-N11-N7** | 114.168 |
| **N11-N7-N3** | 100.333 |
| **N7-N3-N1** | 114.169 |
| **N4-N3-N7** | 122.173 |
| **N8-N11-N7** | 122.173 |

**Table S3.** Torsion angles of ***m*-2** (^o^)

| **N2-N1-N3-N7** | 0.834 |
| --- | --- |
| **N1-N2-N11-N7** | 0.835 |
| **N4-N3-N7-N11** | 173.658 |
| **N8-N11-N7-N3** | 173.656 |

**Table S4**. The calculation of BDE of compounds ***m*-2**

|  | ·NH_2_·/a.u | ·N_5_-NH_2_·/a.u | N_5_-2NH_2_·/a.u | BDE /kJ mol^−1^ |
| --- | --- | --- | --- | --- |
| N-NH_2_ | -55.808976 | -328.846332 | -384.785883 | 342.82 |

**References**

1. Xu, Y. G. et al. A series of energetic metal pentazolate hydrates. *Nature* **549**, 78-81 (2017).
2. Frisch, M. J. et al. Revision E.01, Gaussian Inc., Wallingford CT (2013).
3. Politzer, P., Martinez, J., Murray, J. S., Concha, M. C. & ToroLabbe, A. An electrostatic interaction correction for improved crystal density prediction. *Mol. Phys.* **107**, 2095-2101 (2009).
4. Politzer, P., Martinez, J., Murray, J. S. & Concha, M. C. An electrostatic correction for improved crystal density predictions of energetic ionic compounds. *Mol. Phys.* **108**, 1391-1396 (2010).
5. Rice, B. M., Hare, J. J. & Byrd, E. F. C. Accurate predictions of crystal densities using quantum mechanical molecular volumes. *J. Phys. Chem. A* **111**, 10874-10879 (2007).
6. Jenkins, H. D. B., Roobottom, H. K. & Passmore, J. Relationships among ionic lattice energies, molecular (formula unit) volumes, and thermochemical radii. *Inorg. Chem.* **38**, 3609-3620 (1999).
7. Jenkins, H. D. B., Tudela, D. & Glasser, L. Lattice potential energy estimation for complex ionic salts from density measurements. *Inorg. Chem.* **41**, 2364-2367 (2002).
8. Kamlet, M. J. & Jacobs, S. J. Chemistry of detonations. I. A simple method for calculating detonation properties of C–H–N–O explosives. *J. Chem. Phys.* **48**, 23-35 (1968).
9. Dong, K. et al. Formyl azido substituted nitro hexaazaisowurtzitane–synthesis, characterization and energetic properties. *New J. Chem.* **37**, 3685-3691 (2013).
